# Dividing Professional Fees

**Published:** 1910-04

**Authors:** Matthew D. Mann

**Affiliations:** Buffalo, N. Y.


### BUFFALO MEDICAL JOURNAL.

VOL. LXV. APRIL, 1910. No. 9

### ORIGINAL COMMUNICATIONS.

### Dividing Professional Fees[1]

BY MATTHEW D. MANN, A. M., M. D., Buffalo, N. Y.

THIS is a commercial age. To many minds, money seems to be omnipotent, and there is a tendency toward its pursuit, either by fair means or foul, as a worthy end in life. The so-called "learned professions" have, up to recent times, withstood this tendency and maintained a high tone, holding themselves above the mere pursuit of wealth as a chief aim, thus putting themselves on a plane far higher than that of the trades or commercial businesses.

The difference between a business and a profession is that one is largely selfish, while the other is, or should be, to a considerable extent, altruistic. A merchant, by collecting wares from various places and countries, makes convenient a larger choice of possible purchases. He may also, in competition with rivals, lower prices, but he never gives away his wares for nothing, except as an advertisement,—his sole and only object being personal gain. With the learned professions, it is different. The man who ministers to our souls, administers the sacraments of the church, and, by his life and teaching, seeks to spread the gospel of peace and love, does so, not solely for pay, but because he is filled with a sense of duty. He must live by his work, but the living afforded him is often meager and pitiful in the extreme; so his reward must come from some other source. If he allows mercenary motives to creep in to modify his actions, his usefulness is lost.

The lawyer who defends us when attacked, who saves our estate, or our liberty, or our life even, when unjustly endangered, renders a service which cannot be estimated in money, and which is often inadequately rewarded. It is his duty to do just as good service for the poor client as for the rich. His aim should be to see justice done, and to aid in the accomplishment of right; and it is when he departs from this rule, that he degrades his profession. So it is with our own calling. It is noble if we

1. Read before the Medical Society of the County of Erie, February 21, 1910.

choose to make it so, but may become one of the most degrading and degraded occupations in which a man may engage when practised from low and mercenary motives.

The great object of the profession of medicine is unselfish: to bring help to suffering humanity, help when attacked by disease, or, better still, help by prevention. Preventive medicine is the climax, the highest function, of the profession. No other profession or calling is so constantly and so successfully striving to withdraw the props which support it. For this we get no pay, and are constantly compelled to contend with opposing forces. Nor is our proper professional work done for pay alone. We never turn away anybody because they cannot pay, but always make some suitable provision for their proper care.

Nevertheless, we must live and live by our profession, for the workman is worthy of his hire. How to regulate our remuneration is a hard matter. "We give something which cannot be weighed or measured." Health, relief from suffering, and life are imponderable; and, yet, it is often for their restoration that we must ask a reward. The amount of this reward must be regulated by the financial ability of the patient. We must be content with what he can fairly give, because no money value can be put on our services.

This makes the practice of medicine a profession and not a business, and sets up a high standard of professional conduct. Unfortunately, this is not the standard of some of our members, and just in so far as they depart from it, do they tend to bring down the profession to the level of a business, in other words, to commercialise it.

This departure from the proper standard is shown in several ways. The most degrading and one likely to be followed by disastrous consequences is a practise which has sprung up within recent times of the secret dividing of fees. A physician—the family doctor, we will say,—has a case which seems to need a surgical operation. Not having a surgical training, he seeks a surgeon of skill and experience to do the work for him. The operation is performed, the patient recovers, the surgeon receives his fee, the doctor is paid. Everything is well, and all are contented. This is the proper or normal method. But, sometimes, and quite commonly of late, the doctor, thinking more of his purse than of the patient's welfare, seeks a surgeon who is willing to divide his fee and to give a part to the doctor. So far has this gone, that surgeons seek to get operations by open competition in the percentage of fees offered the doctors, and doctors hawk their patients around, looking for the surgeon who will give them the largest percentage of the fee, who will, in other words, give them a bribe. Of course this is all done on the quiet, as is usual with bribery.

While the practice of dividing fees, or dichotomy, as it has been called, is comparatively modern, having been originated about 1870, it is said, by Pean, the distinguished French surgeon, history tells us that it was attempted in France even earlier. The story quoted in an editorial in the *Journal of the A. M. A.* (June 20, 1903) is somewhat as follows:

In the time of Louis XIV, a young surgeon in Paris had been very successful, but had not yet been able to add the King to his visiting list. Waiting until his older rival, the surgeon to the King, was out of town, he suggested to the King's physician that he be called to do a phlebotomy. Called he was, and the royal blood was made to flow, and a handsome fee came into his hands. He promptly turned over a part of this to the physician. The King heard of it and was very angry. He promptly banished the physician for trading in the royal blood, and punished the surgeon.

So dichotomy was not popular with Louis at least, and seems to have had a setback at the start. Still, it has been revived, and it is necessary for us to study its effects. Let us see what they are.

### EFFECT ON GENERAL PRACTITIONERS.

A man having the money side of his case alone in view, must be led into many errors. He becomes careless in diagnosis. He jumps at unwarrantable conclusions, and, led by the glitter of prospective gold, rushes his patient off to the hospital for an operation which is, perhaps, unnecessary and unjustifiable. He weakens his own conscience, and changes his feelings toward his patients. Instead of friends to be protected and helped, he gets to look upon them as so many victims to be plucked. The question he asks himself is not, can this patient be helped by good nursing, good advice, and proper medication, but, rather, can I find any excuse for an operation, and so gather in a portion of the fee.

When the operation is needed and is justifiable, instead of honestly advising the surgeon as to the financial condition of the patient, for the benefit of the patient, he urges the surgeon to charge a big fee, assuring him that the patient is well able to pay, when the contrary is perhaps the truth. In both these cases there is a conspiracy to rob the patient, and the question arises, whether, were the facts capable of proof, an action could not be successfully maintained in a court of law.

### THE EFFECT ON THE SURGEON OR SPECIALIST.

He, too, will become careless in diagnosis, accepting the ready-made diagnosis of the doctor who brings the case, seeing only that an operation is possible, and perhaps warranted. He feels obliged to operate, even when no operation is needed, in order to sustain the opinion of the doctor, and so maintain the doctor in the good graces of his patient's friends, and thus secure his adherence to himself, and a future supply of operations. The best interests of the patient are not looked out for. "Surgical judgment," one of the best assets of the good surgeon, is not stimulated, but becomes "pocket-book judgment" and a general demoralisation results. Study and progress are not needed to keep up his reputation. He has a constant supply of operations from his "runners," and that is all he cares for. Time spent in study and research, he looks upon as wasted, and he only laughs at his honest neighbor who burns his midnight oil in an endeavor to keep up his professional knowledge, and patiently waits for his reward.

Perhaps, however, the worst effect is the blunting of his sense of right and wrong, which results in the performance of needless operations and the taking of unnecessary risks. Should a patient die under such circumstances, from some unlooked-for accident, then the surgeon is little better than a murderer, and the doctor an accessory.

Here we come to an explanation of why such a state of things as I have described is possible. It is due, strange as it may seem, to the wonderful advance which has been made in modern surgery. Operations are so safe today, that the risk is little considered. When the abdomen can be opened one hundred times consecutively without a death, as has often been done, a careful diagnosis and good surgical judgment, to a conscienceless man, are of little account. He can make his diagnosis during the operation, and, if nothing wrong is found, the patient is pretty sure to recover. He will have a scar, an empty pocket-book, and a memory of several days of suffering to look back upon, and perhaps an illfounded sense of gratitude to the surgeon who, he wrongly thinks, has saved his life. Some day, perhaps, he will find out the truth, and his opinion change.

### EFFECT ON MEN BEGINNING THEIR PRACTICE.

It is well to consider what effect such a false system of ethics may have on the beginners in the profession. A young man recently told me that he had not been in practice six months before he had visits from three different surgeons. They each offered him a percentage of the fees in any cases he might bring to them. They told him that they knew the early years of practice were often hard, and suggested this as a way of making both ends meet. Leaving a handful of cigars, they departed. The young man so approached will either reject the proffered bribe with scorn, or else will allow the influence to have its expected effect. If poor, he may resist for a while, but, under the stress of poverty and want, gives in and becomes a grafter, like others he knows. It is a great temptation to put in a young man's way.

EFFECT ON THE YOUNG MAN WANTING TO ENTER THE PROFESSION.

On finding out that such a state of things exists in the medical profession, a highminded man will turn his back on it in disgust. He sees that he will be unable to compete with grafters, and so will have none of it. If of another moral stamp, he sees his chance, and goes into the profession with false ideas and ideals, and joins the grafters. Thus, there is danger that the profession will be continually recruited from the lower classes, lower morally, at least,—which must result in its ultimate degradation.

EFFECT ON THE PATIENT.

But what of the patient? By this plan the doctors conspire to operate on him needlessly and to rob him. He gets poor surgical service, because the less skilful and the less experienced the surgeon, the more he needs patients, and the larger percentage he will pay. He is the one, therefore, to be chosen by our commercial doctor.

The patient has to pay more than he otherwise would, because the surgeon has to charge a fee large enough to enable him to divide, and he is stimulated to do so by the doctor, who expects his percentage. Moreover, as this division is always done in secret, the poor patient probably has to pay a large bill to the doctor when he gets home, ignorant that the doctor already has had a big slice of the fee paid the surgeon.

If the surgeon can afford to do his work for less than the apparent price, then he is charging too much. As there is a premium on faulty diagnosis and inexperienced operating, the patient perhaps suffers unnecessarily, has a poor chance for recovery and a cure, and perhaps equally unnecessarily loses his life. If he finds out the collusion between the surgeon and the doctor, he loses his respect for both the surgeon and doctor, and for the profession as a whole.

EFFECT ON THE GENERAL PUBLIC.

This loss of respect for the profession when these facts—and they are facts—become known, will have disastrous effects, both on the public and on the profession. The public, fearing both the doctor and surgeon, will put off necessary operations until too late, and so lives will be needlessly sacrificed. Losing faith in the regular profession, and not recognising that it still contains many honest members, or not knowing how to find them, they will turn to some of the popular fads, like Eddyism and osteopathy, to the detriment of both the public and the profession.

I think it will be admitted that this secret division of fees ought to be stopped. Before we discuss the methods by which this may be brought about, let us examine the excuses given by those who uphold this practise, for there are members of the profession who maintain that it is right and fair.

The advocates claim, both the surgeon and the doctor, that the doctor is ordinarily not adequately paid; that a good diagnosis of some obscure condition demanding immediate operation to save life, is quite as important, and should be as well recompensed, as the operation itself. There is doubtless truth in this. but such cases make up a very small part of a surgeon's practice. Physicians are often not well paid under such circumstances. But whose fault is it? Clearly their own. for not insisting on their rights. They should not refrain from making a demand for a proper fee, leaving it to the surgeon to double his fee and give them half. This is merely attempting to cure one evil by another. The public needs educating. If there is any doubt about the doctor's being well paid, then the surgeon certainly should openly help him to get his rights. This is often successfully done, and is a very different thing from the habitual secret dividing of fees. Let the surgeon openly tell the patient, or his friends, that the fee he is collecting is for both,—namely, a joint fee, to be divided.

The *Progrés Médical* cites a suggestion of a certain medical syndicate as laudable,—that the family physician should get one-fourth as much as the surgeon; but no such hard-and-fast rule can be made. It must depend on circumstances.

The claim that the doctor is unable to collect his own fee is no argument for a secret division of the fee. It is pleading the baby-act for the doctor, and no self-respecting man would desire such an argument used in his behalf. It is sophistical and wrong. Let the doctor stand up for his own rights, and let the surgeon openly help him in obtaining them. if he has any difficulty.

After, all, the surgeon need not be so solicitous about the rights of the poor doctor. Sometimes it happens that the surgeon is cheated out of his rights by an unscrupulous doctor. The doctor collects the fee and takes a large portion for himself, telling the surgeon that the patient is too poor to pay more. This and other "games" are often worked on the "poor" surgeon. I could relate many experiences of this kind, which go to show that most general practitioners are quite able to look out for themselves, and that the surgeon has need to keep his eyes open to obtain his rights.

The practice seems to be extending in unlooked-for directions. Physicians (not surgeons), doing a consulting practice, have informed me that they have been "held up," and asked to divide their consultation fee, threats being made of no more consultations from that quarter unless this demand was acceded to. This may lead to quite as bad results as the dividing of purely surgical fees.

Another way in which it is tried to cover up or make right the dividing of fees, is by employing the doctor as assistant at the operation, and secretly, sometimes openly, paying him for his services far above their value. This, again, is all wrong. All surgeons of any prominence or reputation should have their regular assistants, who understand their methods and are of great value in securing good results. To take up with a poor assistant, without experience or training, results in slovenly work, more time employed in the operation, and a lessened chance for the patient. Every surgeon knows this, and appreciates what a good assistant does for him. The argument in favor of this procedure is fallacious, and the practice a mere subterfuge or evasion.

A plan adopted by some is to make no agreement or promise, but when the operation is completed and the fee paid, the surgeon sends a check for as much as he thinks proper. The doctor accepts it in silence. In other words, it is a hands-behind-your-back affair. This surgeon's custom becomes known, and his practice increases as the knowledge spreads. This, it seems to me, is a sneaky, underhand way of doing things, and is the worst of all.

Another plan is to allow the family physician to give the anesthetic, and then for the surgeon to pay him privately an amount out of proportion to the services rendered. This may not work harm to the patient, if the doctor is a skilled anesthetist; but the chances are that he will know little about the giving of either ether or chloroform, and thus the life of the patient will be wrongfully, and often needlessly, endangered.

Unquestionably, this practice of fee-splitting has been made more prominent of late by the financial depression and by the overcrowding of the profession. The medical schools have been blamed for this, but this is quite evidently unfair, as the practice is just as common and the overcrowding just as great in cities where no medical school exists.

I have thus attempted to show that the practice of secretly dividing fees between the attending physician and the operating surgeon is subversive of the best interests of all parties concerned. It is demoralizing the profession. It is causing unnecessary operations, thus subjecting patients to paying unnecessary fees, and to the added risk of life and health. It will certainly cause a loss of confidence in the profession and thus tend to boost irregular methods of practice. It will make it more difficult to prevail upon patients to submit to necessary operations, and thus cause many deaths. It will keep out many able and right-minded young men and fill the profession with those of a lower moral standard.

Undoubtedly a reform is necessary in regard to medical fees. A good diagnosis, which results in saving life by immediate surgical interference, should be well paid. The reform must start from the doctor, and not from the surgeon.

Let us see what others have said about secret dichotomy. There is already a considerable literature to draw from.

The American Medical Association, in its *"Principles of Medical Ethics,"* declares, Art. VI, Sec. 4;

It is derogatory to professional character for physicians to pay or offer to pay commissions to any persons whatsoever, who may recommend to them patients requiring general or special treatment or surgical operations. It is equally derogatory to professional character for physicians to solicit or to receive such commissions.

In Belgium the Council of the College of Physicians promulgated a decree that:

A physician may not charge a commission of a surgeon to whom he sends a case, and no surgeon may allow such a fee.

An editorial in the *Journal A. M. A.* (loc. cit.) says:

With us it (dichotomy) has certainly never had even a taint of respectability about it and has been practised only by those who travel on the ragged edge of regularity and those who are clear over it, among the outlaws of the profession.

Dr. Bacon Saunders, of Fort Worth, Texas, calls it a "profession-degrading, conscience-searing, bargain-counter deal." He says it is due to thoughtlessness, or if not due to that, is due to avaricious cupidity and cannot be defended by any honorable right-thinking man. "If there is any class of men on earth," he says, "whose mainspring of action should be the highest conception of professional, social and moral rectitude, it is our profession."

Dr. A. A. Eshner, Philadelphia (*Bull. A. A. of Med.,* December, 1909) says:

Such a course would be compromising to both parties to the transaction, and it could result only in the demoralization of both donor and recipient and the eventual commercialisation of medicine.

Again:

What has been said with respect to the relations among physicians, applies equally to druggists, instrument-makers, and other tradesmen. The payment and acceptance of commissions for articles purchased by patients merely puts an added burden on the latter and entirely without their knowledge and consent.

Dr. E. Gard Edwards (loc. cit.) says:

Not many months ago, it was considered news to such an extent that several daily (German) papers made half-column mention of the scandal of 'brokerage on patients,' which involved many of the shining lights of the German profession.

Again:

Come as it may, clothed as it is in all manner of garb, fee-splitting is graft, pure and simple, whenever the general practitioner accepts a fee for the simple reference of a case, even when it is done in the guise of expenses paid, because the latter has, as a matter of fact, done nothing for the patient. It is a debasing practice, which tends to supply an unworthy man with patients, simply because he will pay commission to the patient's financial, and, possibly, material detriment.

Dr. Casey A. Wood, of Chicago, says:

There ought not to be any division of fees. Every departure from this commonsense arrangement is fraught with danger to the best interests of the profession, the family physician, the consultant and the patient.

Dr. J. B. Roberts, of Philadelphia, says:

I cannot see how any honorable doctor, preacher, or lawyer could ever believe that a secret division of a fee were a professional propriety, yet some doctors have evidently entertained such a belief.

Dr. William F. Metcalf in his presidential address before Detroit Academy of Medicine says:

Here has arisen the most cloven-footed abuse that threatens the integrity of medical practice today. I refer to the 'division of fees;' by which the general practitioner obtains from the patient, without the patient knowing it, a fee in the form of a gratuity paid out of the specialist's fee and one which he is not honest enough to ask from the patient on his own account. The possibilities of this abuse are infinite at the hands of the no less dishonest specialist, consultant, or surgeon, unless an unmistakable protest by the self-respecting part of the profession goes out against it. The people of this country have supposed they were rid of the buying and selling of human beings; but what else is it when a man in general family practice, by referring a patient to one specialist, is assured of his velvet reward, while if he permits that same patient to go to an honest consultant, the graft game is not possible? Either party to this kind of compact will naturally regard less and less any interest but his own desire for immediate gain. The vital features of this situation are not affected by any excuse that others are doing the same thing. As individuals, we must be willing to make any sacrifice to maintain our honor and refuse to degrade those to whom such a course is tempting. Medical societies should deal with the evil in the severest manner; and the medical and lay boards of institutions can exercise a large influence by refusing privileges to men addicted to this practise.

In fact, while I have described this as a growing evil, it must in justice be said that the bulk of our profession has spotted it for just what it is, and the man who continues the practice will become an outcast before his fellows. It only remains for the people of any intelligent community to know the facts, to hasten this conclusion.

We can respect the porter who not only expects his *tip* and is not ashamed to be seen taking it, but not the doctor who sells his patients and their confidence.

I wonder how the fee-splitters like being put on a level with porters, barbers, waiters and bellhops.

How can this practice be stopped? I have given this matter much thought, and have come to these conclusions:

First—This (Erie County) Society must take the matter in hand. Resolutions must be passed condemning the matter. The society must put itself clearly on record and establish a positive standard. If necessary, those known to be doing it,—and positive proof can be brought against some of the leading men in town,—should be forced to sign an agreement that they will do it no more.

The county societies in the neighborhood must do the same thing, for it is from the country doctor that some, by no means all, the grafting doctors come. I call upon the county societies adjoining Erie County to act, and to act strongly. I hope also that the State and National Associations will give this matter careful consideration and take definite action. This practise is not confined to Erie County or New York state, it is widespread and is growing. Now is the time to stop it.

Hospital boards can also take action. The surgeons pursuing this practice, are mostly members of the hospitals staffs. I call upon the authorities of the Buffalo hospitals, in which the medical or surgical staff is carrying on the practice of secretly dividing fees, to take action at once, and to cause them to stop it, or else to expel the offenders.

Lastly, we must arouse public opinion, both within the profession and outside. This I hope to do hereabouts by the publication of this paper, and I trust I shall have the aid and assistance of all the honest members, and this, fortunately, means a large majority, of the profession. The laity will help when they know the truth, for most people will feel as did Louis XIV, when they find that their lives or health is being offered for sale on the bargain counter.

Our profession has done too much good, is too high and too noble to be trailed in the dust by men of a low moral standard. Let us act together as men in this matter, and stand together for reform.

37 Allen Street.

